# Phytochemical profiling and *in silico* evaluation of *Artemisia absinthium* compounds targeting Leishmania N-myristoyltransferase: molecular docking, drug-likeness, and toxicity analyses

**DOI:** 10.3389/fchem.2024.1508603

**Published:** 2024-11-28

**Authors:** Farouk Boudou, Amal Belakredar, Alaeddine Berkane, Ahcen Keziz, Huda Alsaeedi, David Cornu, Mikhael Bechelany, Ahmed Barhoum

**Affiliations:** ^1^ Department of Biology, Faculty of Sciences, Djillali Liabes University of Sidi-Bel-Abbes, Sidi-Bel-Abbes, Algeria; ^2^ Department of Biotechnology, Faculty of Natural Sciences and Life, University of Mostaganem Abdelhamid Ibn Badis, Mostaganem, Algeria; ^3^ Laboratory of Chemistry, Synthesis, Properties, and Applications (LCSPA), Department of Chemistry, Faculty of Sciences, Dr. Moulay Tahar University of Saida, Saida, Algeria; ^4^ Physics and Chemistry of Materials Lab, Department of Physics, University of M’sila, M’sila, Algeria; ^5^ Department of Chemistry, College of Science, King Saud University, Riyadh, Saudi Arabia; ^6^ Institut Européen des Membranes, IEM, UMR-5635, University Montpellier, École Nationale Supérieure de Chimie de Montpellier (ENSCM), Centre National de la Recherche Scientifique (CNRS), Montpellier, France; ^7^ Functional Materials Group, Gulf University for Science and Technology (GUST), Mubarak Al-Abdullah, Kuwait; ^8^ NanoStruc Research Group, Chemistry Department, Faculty of Science, Helwan University, Cairo, Egypt

**Keywords:** *Artemisia absinthium*, Leishmania, N-myristoyltransferase, molecular docking, molecular dynamics

## Abstract

**Background:**

*Artemisia absinthium* has long been recognized for its therapeutic properties against various diseases. Among these is leishmaniasis, a parasitic infection that remains a global health challenge. Targeting Leishmania N-myristoyltransferase (NMT), a crucial enzyme for parasite survival, represents a promising therapeutic approach. The bioactive compounds in *A. absinthium* could potentially inhibit NMT and serve as new treatment options for leishmaniasis.

**Aim:**

This study aims to investigate the phytochemical composition, drug-likeness, and molecular dynamics of *A. absinthium* bioactive compounds targeting Leishmania NMT, identifying potent inhibitors that could serve as new drug candidates.

**Method:**

The extract of *A. absinthium* was analyzed using High-Performance Liquid Chromatography (HPLC), identifying nine phenolic compounds, with kaempferol (10.72%) and chlorogenic acid (4.43%) being the most abundant. Drug-likeness and toxicity were evaluated using SwissADME and OSIRIS Property Explorer, focusing on adherence to Lipinski’s rule of five and Ghose’s filter. Molecular docking studies were conducted to evaluate the binding affinity of these compounds to NMT. Molecular dynamics (MD) simulations were performed to assess the stability and flexibility of the NMT-apigenin complex.

**Results:**

Molecular docking identified apigenin as the most potent NMT inhibitor, with a binding energy of −9.6 kcal/mol, forming significant hydrogen bonds with threonine residues 203 and 189. Drug-likeness analysis revealed that most compounds adhered to Lipinski’s rule of five, indicating favorable pharmacokinetic properties. MD simulations confirmed the stability of the NMT-apigenin complex, with root mean square deviation (RMSD) values of 0.04 nm, root mean square fluctuation (RMSF) values between 0.05 and 0.35 nm, and radius of gyration (Rg) values ranging from 2.24 to 2.30 nm. Normal mode analysis further supported the complex’s stability and flexibility.

**Conclusion:**

The findings of this study underscore the potential of Artemisia absinthium compounds, particularly apigenin, as promising candidates for the development of new anti-leishmaniasis drugs. The potent inhibition of Leishmania NMT by apigenin, along with its favorable pharmacokinetic and stability profiles, supports its further exploration in antileishmanial drug development.

## 1 Introduction

According to the World health Organization, leishmaniasis is one of the seven most important tropical diseases, and a severe global health threat due to its potentially lethal symptoms (Torres-Guerrero et al., 2017). This parasitic disease is caused by protozoan parasites from the Trypanosomatida order and is transmitted through the bite of infected sandfly species, primarily *Phlebotomus* and *Lutzomyia*. These vectors are prevalent in tropical and subtropical regions, such as Europe, North Africa, the Middle East, and parts of South America (Vera-Izaguirre et al., 2006). Depending on the *Leishmania* species and the host immune response, the disease can present different clinical forms, ranging from the disfiguring ulcerative skin lesions of cutaneous leishmaniasis to the life-threatening visceral leishmaniasis (Andrade-Narváez et al., 2001; Sunyoto et al., 2018). *Leishmania tropica*, *Leishmania Leishmania major* and *Leishmania Leishmania aethiopica* cause Old World cutaneous leishmaniasis, whereas *Leishmania Leishmania mexicana* and *Leishmania Leishmania braziliensis* cause New World cutaneous leishmaniasis, particularly in Mexico and Central America (Gabriel et al., 2019). Despite leishmaniasis high prevalence, especially in regions such as Afghanistan and Algeria (Benikhlef et al., 2021), the current treatments face significant challenges, including high toxicity, low efficacy and the emergence of drug resistance, underscoring the urgent need of novel therapies (Sang et al., 2018; Pinheiro and de Souza, 2022).

Due to the chemical variety and bioactive characteristics, natural products derived from medicinal plants are interesting and promising compounds for drug discovery (Sasidharan et al., 2011; Balakrishnan et al., 2020). More than 80% of the global population relies on traditional medicine for primary healthcare, and medicinal plants have long been a source of inspiration for the development of new drugs (Shanker et al., 2014). For instance, plant-derived compounds have been used as the basis for anti-malarial drugs, such as artemisinin and quinine. *Artemisia absinthium*, commonly known as wormwood, is a medicinal plant with a long ethnobotanical history, traditionally used in Europe, Asia, and North Africa to treat various ailments, including parasitic infections. Despite its widespread use in traditional medicine, data on *A. absinthium*’s antileishmanial potential remains limited (Hussain et al., 2017). Its rich phytochemical profile, including compounds such as flavonoids, terpenoids and phenolic acids, suggests that *Artemisia abstinthium* could be a source of novel therapeutic agents, particularly for diseases like leishmaniasis for which current treatment options are inadequate (Batiha et al., 2020).

N-myristoyltransferase (NMT) is an essential enzyme for Leishmania lifecycle and a validated target for antileishmanial drug development. NMT is involved in the post-translational modification of proteins by attaching a myristoyl group to their N-terminal glycine residue, a process crucial for the parasite survival and virulence. As NMT inhibition disrupts multiple biological pathways in the parasite, this enzyme an attractive target for drug discovery. Recent advancements in computer-aided drug discovery allow exploring natural product libraries to identify potential NMT inhibitors. Molecular docking simulations, a key tool in computer-aided drug discovery, are used by researchers to model the interactions between small molecules and target proteins, enabling the identification of potential inhibitors (Dias et al., 2008; Sliwoski et al., 2014). The finding that plant-derived compounds, particularly flavonoids and terpenoids, exhibit inhibitory activity against various parasitic enzymes (Mukherjee et al., 2016) suggesting that *A. absinthium* also may contain phytochemicals that act as NMT inhibitors.

The aim of this study was to investigate *A. abstinthium* antileishmanial potential through a multifaceted approach that involved both phytochemical and computational methodologies. First, high-performance liquid chromatography (HPLC) was employed for the in-depth quantitative profiling of the *A. absinthium* extract. This allowed us to identify and quantify key bioactive compounds, including flavonoids, phenolic acids and terpenoids. Then, rigorous computational analyses, including molecular docking simulations, were used to assess their binding affinity and inhibitory potential against Leishmania NMT. This dual approach (phytochemical profiling and advanced *in silico* techniques) marks a novel contribution to the field. To our knowledge, this is the first comprehensive investigation of *A. absinthium* compounds to target NMT for antileishmanial activity. The novelty of this study lies in its integrated methodology that allowed identifying bioactive compounds and provided insights into their potential mechanism of action at the molecular level. This innovative approach enhances the understanding of *A. absinthium*’s therapeutic potential, offering valuable insights for the development of new, effective treatments for leishmaniasis, a disease with limited treatment options and a high global burden.

## 2 Experimental

### 2.1 Plant extraction and phytochemical profiling

To prepare the extract, 10 g of dried and crushed *A. absinthium* leaves were macerated in 100 mL of methanol for 48 h. During the extraction process, the solvent was refreshed after 24 h to maintain the extraction efficiency and the mixture was stirred magnetically to ensure uniform mixing. After maceration, the mixture was filtered through Whatman No. 1 filter paper, and the resulting filtrate was concentrated by solvent evaporation under reduced pressure using a rotary evaporator. This method followed the guidelines outlined by (Sidaoui et al., 2016). The dried extract was stored at 4°C for further analysis.

The bioactive compounds present in the *A. absinthium* extract were identified and quantified by HPLC using a Shimadzu Prominence-i LC-2030C 3D HPLC system and a Supelco C18 column (25 cm length, 4.6 mm internal diameter, and 5 μm particle size) maintained at 27°C throughout the analysis. A solvent gradient elution system was employed where the mobile phase consisted of acetic acid (aqueous solution) and methanol. The flow rate was set at 0.8 mL/min. Phenolic compounds in the sample were detected by measuring their absorbance at wavelength of 280 nm, allowing the accurate identification of the major bioactive compounds in the *A. absinthium* extract.

### 2.2 Exploration of potential ligands

The therapeutic potential of the compounds present in the *A. absinthium* extract was explored based on the extensive literature data on plant-derived compounds against parasitic infections (Adamu et al., 2012; Adamu et al., 2014). The two- and three-dimensional (2D and 3D) structures of the identified compounds were retrieved from publicly accessible drug databases, such as PubChem and SwissADME (Li et al., 2010), that contain structural information and features essential for the computational analysis.

### 2.3 Drug-likeness assessment

The drug-likeness of the identified compounds was evaluated using the SwissADME database (http://www.swissadme.ch). For each compound, The Simplified Molecular Input Line Entry System (SMILES) notations were obtained, and if unavailable, they were drawn using ChemAxon’s Marvin JS molecular sketcher. Their physicochemical properties and pharmacokinetic characteristics were analyzed, with a specific focus on compliance with the Lipinski’s Rule of Five (Lipinski et al., 1997) and the Ghose filter (Ghose et al., 1999). Lipinski’s Rule of Five evaluates drug-likeness based on four key criteria: no more than five hydrogen bond donors, no more than ten hydrogen bond acceptors, a molecular mass ≤500 Da, and an octanol-water partition coefficient (log *P*) ≤ 5. Compounds that comply with these criteria were considered to have a high probability of oral bioavailability. Additionally, the Ghose filter, which imposes further restrictions on molecular size and polarity, was used to refine the compound’s drug-likeness. Radar plots generated by SwissADME visually represented the following physicochemical properties of the compounds: lipophilicity, molecular size, polarity, solubility, flexibility and saturation. The compounds’ blood-brain barrier (BBB) permeability and gastrointestinal absorption were predicted using Boiled-Egg plots.

### 2.4 Target protein selection and molecular docking

For the compounds that exhibited favorable drug-likeness profiles, molecular docking studies were performed to assess their inhibitory potential against *Leishmania*-NMT, an enzyme crucial for the parasite survival and virulence (Satyendra et al., 2015). The crystal structure of *Leishmania* NMT (PDB ID: 6QDF) was obtained from the RCSB Protein Data Bank (www.rcsb.org). The 3D structures of the selected compounds (i.e., the ligands) were retrieved from PubChem (www.pubchem.ncbi.nlm.nih.gov).

Molecular docking simulations were performed using PyRx-Python Prescription 0.8 (Trott and Olson, 2010) to predict the binding interactions between the ligands and NMT active site. Before docking, the enzyme was prepared with the Molegro 2.5 software. The docking results were visualized with Molegro Molecular Viewer version 2.5 that provided detailed insights into the binding affinities and interaction patterns between ligands and target protein. The protein surface topography was thoroughly analyzed using the CASTp server (http://sts.bioe.uic.edu/castp/), to identify potential activation sites and critical binding pockets.

### 2.5 Molecular dynamics simulations

To validate the docking results and assess the dynamic stability of the NMT ligand complexes, MD simulations were carried out using the GROMACS 2023-GPU package with the CHARMM-36-2019 set of force fields. The complexes were solvated using TIP3P water models in a cubic box and neutralized by adding Na^+^ and Cl^−^ ions. Energy minimization was performed to eliminate steric clashes and optimize the system geometry. Subsequently, equilibration was carried out: first in constant volume (NVT simulations) and then in constant pressure conditions (NPT simulations) each for 2 nanoseconds. The final model of the molecular dynamic simulation was run at a constant temperature of 300 K and a pressure of one bar for 250 nanoseconds.

Throughout the simulation, trajectory files were generated and analysed to calculate the root mean square deviation (RMSD), root mean square fluctuation (RMSF), radius of gyration (Rg), and binding free energy calculations (D) that provide insights into the structural stability and flexibility of the NMT ligand complexes.

To further explore the dynamic behaviour of the complexes, the iMODS server (http://imods.chaconlab.org) was used to perform normal mode analysis. This analysis offered a detailed description of the protein movement patterns, deformability, and stability. The eigenvalues, B-factors, and covariance matrix were also analyzed to predict the potential structural changes and transition pathways within the protein.

## 3 Results and discussion

### 3.1 Chemical profiling using HPLC

**FIGURE 1:**
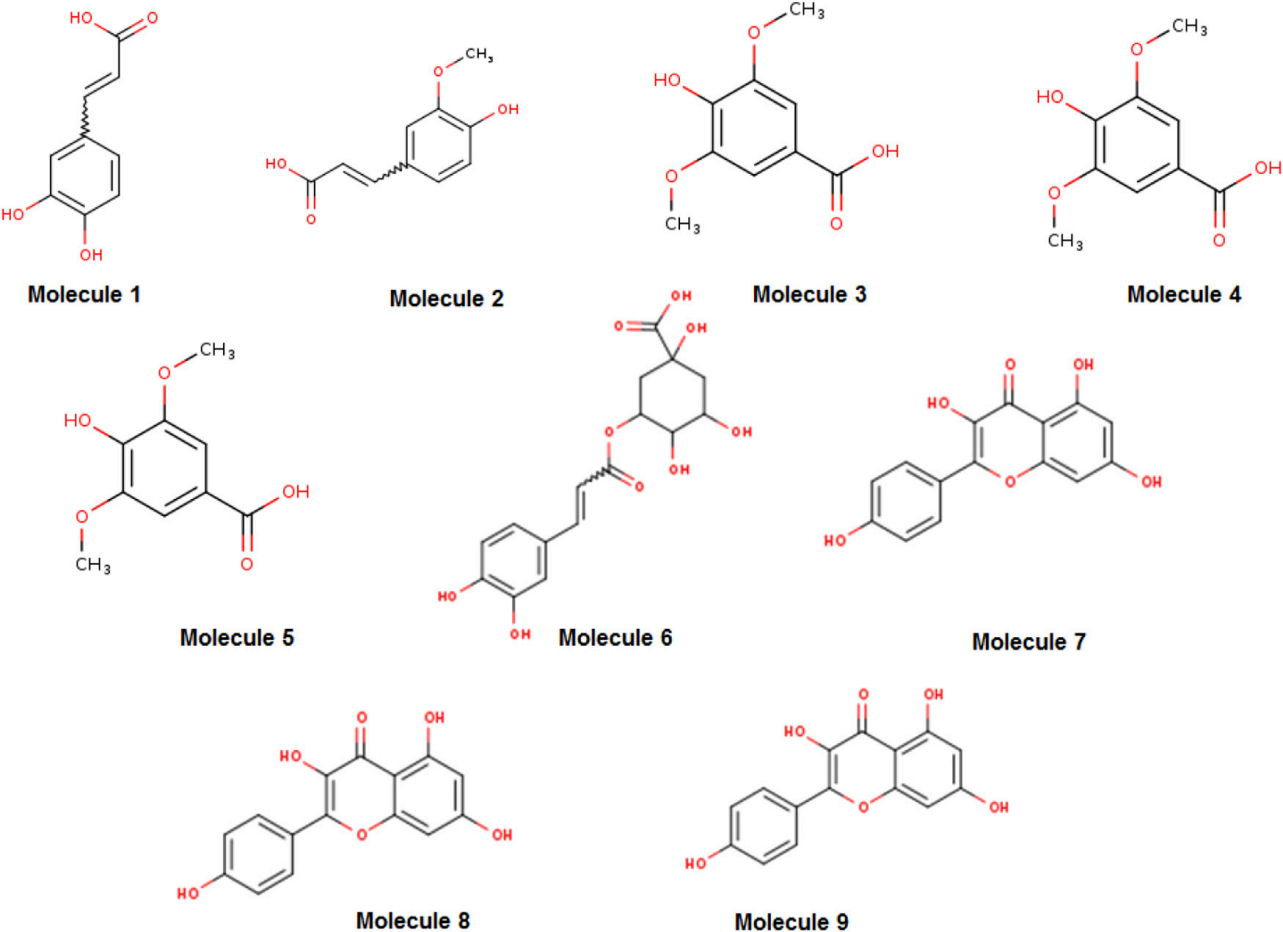
Two-dimensional structures of the main phytochemicals from the *Artemisia absinthium* extract, retrieved from SwissADME. The molecules numbered from one to nine are caffeic acid, ferulic acid, syringic acid, epigallocatechin gallate, vanillic acid, chlorogenic acid, kaempferol, quercetin, and apigenin, respectively.

HPLC analysis of *A. absinthium* extract revealed a rich profile of bioactive compounds, predominantly consisting of phenolic acids and flavonoids, which are well-known for their antioxidant, anti-inflammatory, and anticancer activities. As illustrated in Figure 1 and detailed in Table 1, these findings align with previous studies highlighting the medicinal value of A. absinthium (Batiha et al., 2020; Ali et al., 2021). The most prominent compound identified was kaempferol, constituting 10.72% of the extract. This flavonoid is extensively documented for its significant biological activities, particularly its potent antioxidant and anti-inflammatory properties, which play a critical role in the therapeutic management of chronic diseases such as cancer and cardiovascular disorders (Chen and Chen, 2013). The elevated concentration of kaempferol suggests that *A. absinthium* may serve as a valuable source for developing kaempferol-based therapeutic agents, particularly given its ability to modulate crucial signaling pathways associated with apoptosis and inflammation.

**TABLE 1:** Phytochemical profiling of the *Artemisia absinthium* methanolic extract by high-performance liquid chromatography.

Index	Compound	Retention time (min)	Proportion (%)
1	Caffeic acid	16.71	1.92
2	Ferulic acid	23.19	3.39
3	Syringic acid	25.66	2.15
4	Epigallocatechin gallate	26.96	1.24
5	Vanillic acid	27.49	1.58
6	Chlorogenic acid	28.16	4.43
7	Kaempferol	34.66	10.72
8	Quercetin	35.34	2.09
9	Apigenin	39.95	2.10

In addition to kaempferol, the extract also contained notable concentrations of quercetin and apigenin, measured at 2.09% and 2.10%, respectively. Quercetin is recognized for its extensive health benefits, including anticancer, cardioprotective, and antidiabetic properties (Dabeek and Marra, 2019). Similarly, apigenin is acknowledged for its anti-inflammatory, antioxidant, and anti-tumor activities, making it a promising candidate for therapeutic applications, particularly in cancer prevention and treatment (Salehi et al., 2019). Furthermore, the presence of phenolic acids such as caffeic acid (1.92%), ferulic acid (3.39%), syringic acid (2.15%), and vanillic acid (1.58%) contributes significantly to the medicinal properties of A. absinthium. These compounds exhibit strong antioxidant activities that protect cells from oxidative stress-induced damage. For example, caffeic acid and ferulic acid have been linked to neuroprotective effects and the prevention of neurodegenerative diseases (Zhou et al., 2016). Additionally, chlorogenic acid, which accounted for 4.43% of the extract, is noteworthy for its potential to regulate glucose and lipid metabolism, presenting intriguing possibilities for managing metabolic disorders such as diabetes (Naveed et al., 2018).

Despite being present in lower concentrations (1.24%), epigallocatechin gallate, a potent antioxidant commonly found in green tea, further enhances the extract’s therapeutic potential. This compound modulates various cellular pathways involved in cancer, cardiovascular diseases, and metabolic disorders (Ravindranath et al., 2004). The diverse array of bioactive compounds identified at varying concentrations in A. absinthium suggests a synergistic effect that could amplify their therapeutic efficacy. The array of flavonoids and phenolic acids provides a broad spectrum of biological activities, supporting the traditional use of A. absinthium in treating various ailments. Comparisons with previous studies (Moacă et al., 2019; Elansary et al., 2020) show consistency in identifying key compounds such as caffeic acid, ferulic acid, chlorogenic acid, and quercetin. Variations in the proportions of these compounds across different studies may arise from differences in plant sources, environmental factors, and extraction methods (Salem et al., 2020; Hbika et al., 2022).

The HPLC profile obtained in this study complements previous analyses conducted using GC-MS and LC-MS techniques. GC-MS studies have identified unique compounds, including margaspidin, stigmasterol, and fatty acid esters, such as octadecanoic and hexadecanoic acid derivatives, which exhibit anticancer properties (Sultan and Moni, 2022). Similarly, LC-MS analysis by Singh et al. (2021) on various Artemisia species highlighted major compounds such as kaempferol and artemisinin, reinforcing the therapeutic applications of A. absinthium, particularly due to the high concentration of kaempferol observed in our study. These combined findings underscore the potential of A. absinthium extracts as a natural source for addressing inflammation, cancer, and metabolic disorders. With the increasing challenges posed by conventional treatments, the exploration of bioactive compounds from A. absinthium emerges as a promising avenue for developing effective therapeutic strategies against prevalent diseases.

Traditional antileishmanial drugs, including amphotericin B, miltefosine, pentavalent antimonials (e.g., sodium stibogluconate), and paromomycin, face significant challenges (Olías-Molero et al., 2021; Croft et al., 2006). Although amphotericin B is effective, its high toxicity often leads to renal impairment and infusion-related reactions, limiting its clinical use. Miltefosine, the first oral treatment for leishmaniasis, is easier to administer but is associated with gastrointestinal discomfort and teratogenic risks, particularly for pregnant women, and resistance is emerging in endemic regions (Sundar and Olliaro, 2007). Pentavalent antimonials, long used as first-line therapies, carry severe toxicity risks such as pancreatitis, cardiotoxicity, and hepatotoxicity, requiring careful medical oversight. Given these limitations, there is increasing interest in bioactive compounds from *A*. *absinthium* as alternative treatments for leishmaniasis.

### 3.2 Drug-likeness and bioavailability predictions

Table 2 summarizes the drug-likeness predictions (SwissADME analysis) for the nine compounds from the A. absinthium extract, covering molecular weight (MW), hydrogen bond acceptors (HBA), hydrogen bond donors (HBD), molar refractivity (MR), molecular logarithm of partition coefficient (MLOGP), water logarithm of partition coefficient (WLOGP), and compliance with Lipinski’s rule of five and the Ghose filter.

**TABLE 2:** Drug-likeness predictions of selected herbal compounds from *Artemisia absinthium* using SwissADME. Key physicochemical properties for evaluating drug-likeness, including molecular weight (MW), hydrogen bond acceptors (HBA), hydrogen bond donors (HBD), molar refractivity (MR), predicted partition coefficients (MLOGP and WLOGP), and compliance with both Lipinski’s Rule of Five and the Ghose filter.

Ligands	MW (g/mol)	HBA	HBD	(MR)	MLOGP	WLOGP	Lipinski rule	Ghose filter
Caffeic acid	180.16	4	3	47.16	0.70	1.09	Yes; 0 violation	Yes
Ferulic acid	194.18	4	2	51.63	1.00	1.39	Yes; 0 violation	Yes
Syringic acid	198.17	5	2	48.41	0.49	1.11	Yes; 0 violation	Yes
Epigallocatechin gallate	458.37	11	8	112.06	−0.44	1.91	No; 2 violations: NorO>10, NHorOH>5	Yes
Vanillic acid	168.15	4	2	41.92	0.74	1.10	Yes; 0 violation	Yes
Chlorogenic acid	354.31	9	6	83.50	−1.05	−0.75	Yes; 1 violation: NHorOH>5	No; 1 violation: WLOGP < −0.4
Kaempferol	286.24	6	4	76.01	−0.03	2.28	Yes; 0 violation	Yes
Quercetin	302.24	7	5	78.03	−0.56	1.99	Yes; 0 violation	Yes
Apigenin	270.24	5	3	73.99	0.52	2.58	Yes; 0 violation	Yes

Molecular weight (MW) is an important factor influencing a compound’s absorption, distribution, metabolism, and excretion (ADME). Most of the compounds had molecular weights under 500 Da, aligning with Lipinski’s rule and suggesting favorable drug-likeness, with caffeic acid (180.16 g/mol) and vanillic acid (168.15 g/mol) being the lightest. However, epigallocatechin gallate (458.37 g/mol) stood out for its much larger MW, approaching the upper limit for ideal drug candidates.

Hydrogen bonding (HBA and HBD) influences solubility and permeability. Most compounds demonstrated good balance, with caffeic acid, ferulic acid, and syringic acid having 4 or fewer hydrogen bond acceptors and donors, enhancing their potential for oral bioavailability. In contrast, epigallocatechin gallate and chlorogenic acid had significantly more hydrogen bond donors and acceptors, with epigallocatechin gallate presenting 11 HBAs and 8 HBDs, resulting in two violations of Lipinski’s rule. These excessive hydrogen bonds may negatively impact its membrane permeability and, thus, oral absorption.

Molar refractivity (MR), indicating the compound’s volume and polarizability, was within the ideal range for most compounds, with kaempferol (76.01) and quercetin (78.03) exhibiting high MR values, supporting their suitability for biological interactions. In contrast, epigallocatechin gallate had a significantly higher MR (112.06), indicating a larger molecular size and reduced polarity, which might impact its bioavailability.

Lipophilicity predictions, represented by MLOGP and WLOGP, highlight the compound’s ability to cross lipid membranes. While most compounds fell within optimal ranges, chlorogenic acid had a negative WLOGP (−0.75), violating the Ghose filter and suggesting lower membrane permeability. Conversely, apigenin (WLOGP: 2.58) and kaempferol (WLOGP: 2.28) showed higher lipophilicity, making them more likely to cross cell membranes efficiently.

In terms of overall drug-likeness compliance, most compounds, including caffeic acid, ferulic acid, quercetin, and apigenin, adhered to both Lipinski’s rule and the Ghose filter. However, epigallocatechin gallate did not meet all criteria, with violations in hydrogen bond donors and acceptors, while chlorogenic acid did not pass the Ghose filter due to its low WLOGP, implying lower oral bioavailability compared to the other compounds.

**FIGURE 2:**
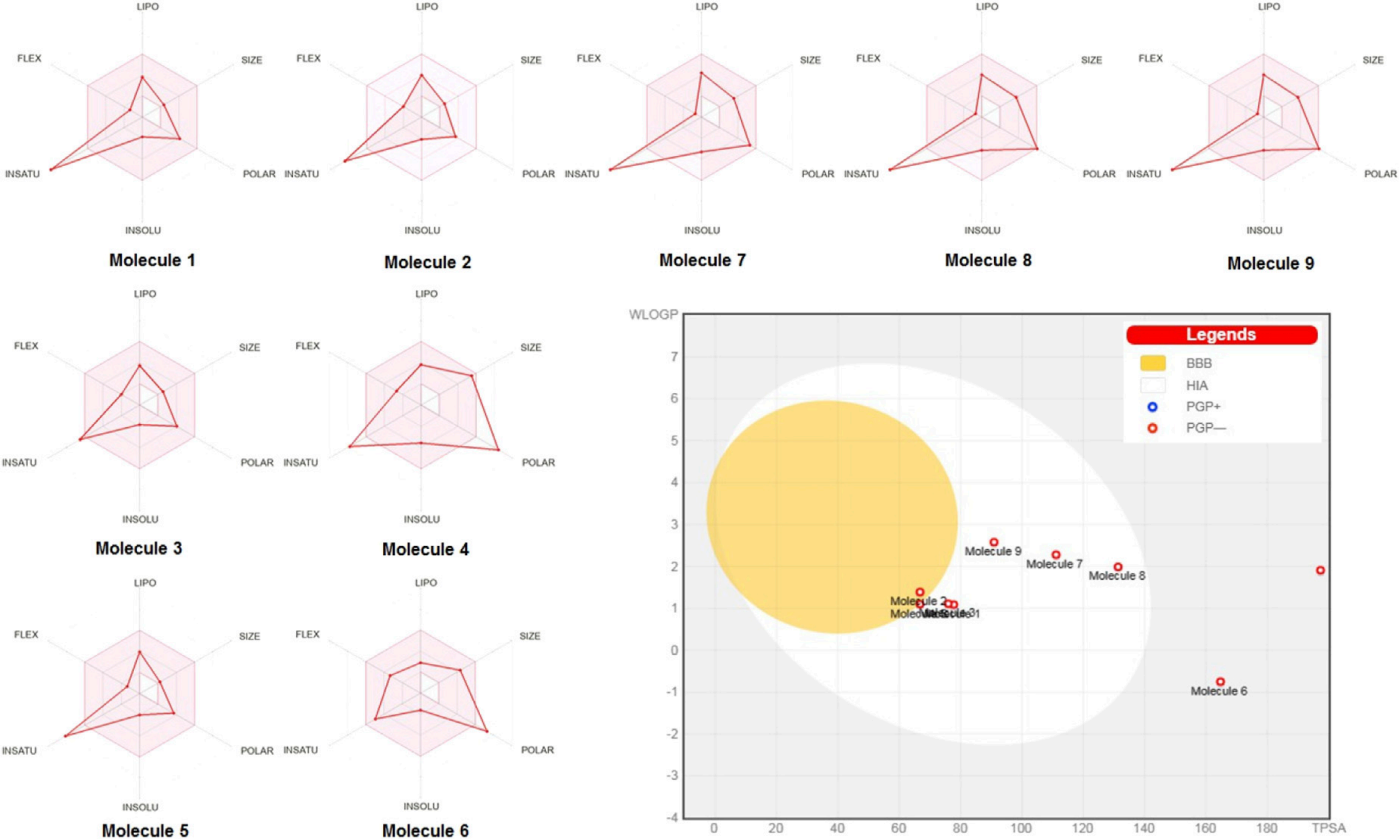
SwissADME radar charts and BOILED-Egg plot for the nine studied molecules (1–9): caffeic acid, ferulic acid, syringic acid, epigallocatechin gallate, vanillic acid, chlorogenic acid, kaempferol, quercetin, and apigenin.

Radar charts (Figure 2) further supported these findings, showing that key pharmacokinetic properties, such as lipophilicity, size, polarity, solubility, saturation, and flexibility, of most compounds were within acceptable ranges for efficient oral absorption. The BOILED-Egg plot revealed potential blood-brain barrier (BBB) penetration and intestinal absorption. Ferulic acid and vanillic acid displayed good potential for BBB penetration, whereas epigallocatechin gallate and chlorogenic acid showed lower potentials for both intestinal absorption and BBB penetration.

Comparisons with previous studies by Shady et al. (2022) and Tian et al. (2015) further underscore the consistent drug-like properties of compounds like caffeic acid, ferulic acid, and quercetin, confirming their suitability as promising drug candidates across different plant sources.

### 3.3 Toxicity assessment of the herbal compounds

The toxicity assessment of the selected herbal compounds, based on key toxicological parameters (mutagenic, tumorigenic, irritant, and reproductive effects), provided a comparative analysis of their safety (Table 3). Using the OSIRIS Property Explorer, the compounds under study were ranked according to their toxicity and drug-likeness and drug scores. The drug score summarizes in a single value the drug-likeness, ClogP, LogS, molecular weight and toxicity risks of a compound.

**TABLE 3:** Summary of the predicted toxicity risks, drug-likeness, and drug score for the compounds extracted from *Artemisia absinthium* extract, assessed using OSIRIS Property Explorer.

Compound	Mutagenic risk	Tumorigenic risk	Irritant risk	Reproductive risk	Drug-likeness	Drug score
Caffeic acid	Green	Green	Green	Green	1.62	0.89
Ferulic acid	Green	Green	Green	Green	1.12	0.84
Syringic acid	Red	Green	Green	Green	1.99	0.44
Epigallocatechin gallate	Green	Green	Green	Green	1.58	0.70
Vanillic acid	Red	Green	Green	Green	1.31	0.35
Chlorogenic acid	Green	Green	Green	Green	0.17	0.70
Kaempferol	Red	Green	Green	Green	0.90	0.46
Quercetin	Red	Red	Green	Green	1.60	0.30
Apigenin	Red	Green	Green	Green	1.21	0.47

Red = high risk of harmful effects; Green = favorable property for drug confirmation.

Caffeic acid and ferulic acid displayed low toxicity risks as indicated by the green indicators for mutagenic, tumorigenic, irritant, and reproductive effects, suggesting favorable safety profiles. Caffeic acid had higher drug-likeness (1.62) and drug (0.89) compared with ferulic acid (1.12 and 0.84, respectively), suggesting a slightly better therapeutic potential. Nevertheless, both compounds were considered safe and promising for drug development due to their minimal toxicity risks.

Syringic acid, showed a potential mutagenic risk, but maintained a low overall toxicity risk in the other categories and had a high drug-likeness score (1.99). However, its lower drug score (0.44) indicated that the mutagenicity risk may limit its therapeutic application without further modifications to address this issue.

Epigallocatechin gallate and vanillic acid showed low toxicity risks, with green indicators in all categories except for the mutagenic risk of vanillic acid. Epigallocatechin gallate had a higher drug score than vanillic acid (1.58 vs. 1.31), indicating moderate drug-likeness and safety for the first and, some limitations in therapeutic applications, possibly due to the mutagenicity risk, for the second.

Chlorogenic acid had a very low drug-likeness score (0.17), indicating limited therapeutic potential despite its low toxicity (all green indicators). Kaempferol and apigenin displayed mutagenic risk and moderate drug-likeness scores (0.90 and 1.21, respectively). However, the mutagenic risks associated with these compounds suggest that safety evaluations are essential before further development.

Safety concerns were higher for quercetin because it showed both mutagenic and tumorigenic risks (red indicators). Despite its relatively high drug-likeness score (1.60), its overall low drug score (0.30) reflected significant toxicity risks that could hinder its use in drug development.

These findings are consistent with the recent study, by Lokhande et al. (2020) who also highlighted concerns about quercetin mutagenic and tumorigenic risks and relatively lower toxicity kaempferol. The agreement between studies underscores the importance of comprehensive toxicity assessments in drug development to ensure safety and efficacy. The toxicity profile variations among the compounds under study suggest that although many exhibit promising drug-like properties, their safety profiles must be carefully taken into account for their potential therapeutic applications.

### 3.4 Molecular docking of binding energies and molecular interactions

Table 4 presents the binding energies and molecular interactions of the assessed phytochemicals with the active site of NMT, the target protein. Among the nine evaluated compounds, apigenin emerged as the most effective binder, exhibiting the strongest binding affinity with an estimated binding energy of −9.6 kcal/mol. This was followed closely by quercetin and epigallocatechin gallate, both showing binding energies of −8.6 kcal/mol, and caffeic acid, which had a binding energy of −7.4 kcal/mol.

**TABLE 4:** Binding energies and molecular interactions of the identified phytochemicals with the active site of NMT enzyme.

Ligand	PubChem ID	Estimated free energy of binding (kcal/mol)	Hydrogen bonding
Caffeic acid	CID: 689043	−7.4	Ile 185, Thr 189
Ferulic acid	CID: 445858	−4.7	Lys 29, Phe 32, Pro 35
Syringic acid	CID: 10742	−6.0	Ser 330, Tyr 345, Asn 376
Epigallocatechin gallate	CID: 65064	−8.6	Tyr 80, Asn 167, Phe 168, Leu 169, Val 171, Tyr 203
Vanillic acid	CID: 8468	−5.9	Phe 88, Ser 330, Tyr 345, Asn 376
Chlorogenic acid	CID: 1794427	−5.6	Ser 285, Ala 351, Ile 354, Leu 356
Kaempferol	CID: 5280863	−6.3	Lys 412, Gln 415
Quercetin	CID: 5280343	−8.6	Asn 167, Phe 168, Thr 189
Apigenin	CID: 5280443	−9.6	Thr 203, Thr 189

The binding interactions reveal the specific residues involved, which are crucial for the stability of the ligand-protein complex. Apigenin forms significant hydrogen bonds with Thr 189 and Thr 203, indicating a strong interaction with the active site. In contrast, quercetin and epigallocatechin gallate interact with Asn 167, Phe 168, and also share hydrogen bonds with Thr 189. While both of these compounds demonstrate substantial binding, their binding energies suggest slightly less favorable interactions than those observed for apigenin.

### 3.5 Molecular docking of binding energies and molecular interactions

Table 4 presents the binding energies and molecular interactions of the assessed phytochemicals with the active site of NMT, the target protein. Among the nine evaluated compounds, apigenin emerged as the most effective binder, exhibiting the strongest binding affinity with an estimated binding energy of −9.6 kcal/mol. This was followed closely by quercetin and epigallocatechin gallate, both showing binding energies of −8.6 kcal/mol, and caffeic acid, which had a binding energy of −7.4 kcal/mol.

The binding interactions reveal the specific residues involved, which are crucial for the stability of the ligand-protein complex. Apigenin forms significant hydrogen bonds with Thr 189 and Thr 203, indicating a strong interaction with the active site. In contrast, quercetin and epigallocatechin gallate interact with Asn 167, Phe 168, and also share hydrogen bonds with Thr 189. While both of these compounds demonstrate substantial binding, their binding energies suggest slightly less favorable interactions than those observed for apigenin. This finding aligns with the study by Fonseca-Silva et al. (2016), which highlighted the potent bioactivity of apigenin against Leishmania amazonensis, where the compound induced reactive oxygen species production and mitochondrial dysfunction, ultimately leading to autophagy and cell death. Similarly, Abugri and Witola (2020) demonstrated the anti-parasitic potential of apigenin-7-O-glucoside against Toxoplasma gondii, where it exhibited growth inhibition, particularly when combined with pyrimethamine, showing an additive effect. Both studies emphasize the versatility of apigenin and its derivatives in targeting pathogens by interfering with essential biological functions.

Caffeic acid, although still a promising binder, shows a lower binding affinity compared to apigenin, with a binding energy of −7.4 kcal/mol. It primarily interacts with residues such as Ile 185 and Thr 189, which are less critical compared to the residues targeted by apigenin. The remaining compounds, including ferulic acid, syringic acid, vanillic acid, chlorogenic acid, and kaempferol, exhibit weaker binding affinities, with estimated binding energies ranging from −4.7 to −6.3 kcal/mol. These lower binding energies indicate less effective interactions within the active site of the NMT enzyme. This trend aligns with the findings of Stefaniu and Pirvu (2022), who conducted an *in silico* analysis of 50 polyphenolic compounds and concluded that flavonoids like apigenin consistently exhibit stronger bioactivity and bioavailability than polyphenolic acids such as ferulic and syringic acids. Their study further highlighted that, while phenyl carboxylic acids like vanillic acid and ferulic acid possess bioactivity, flavonoids tend to be more potent natural cell modulators with better bioavailability. This reinforces the superior binding efficiency of flavonoids like apigenin and quercetin compared to the polyphenolic acids evaluated in this study.

**FIGURE 3:**
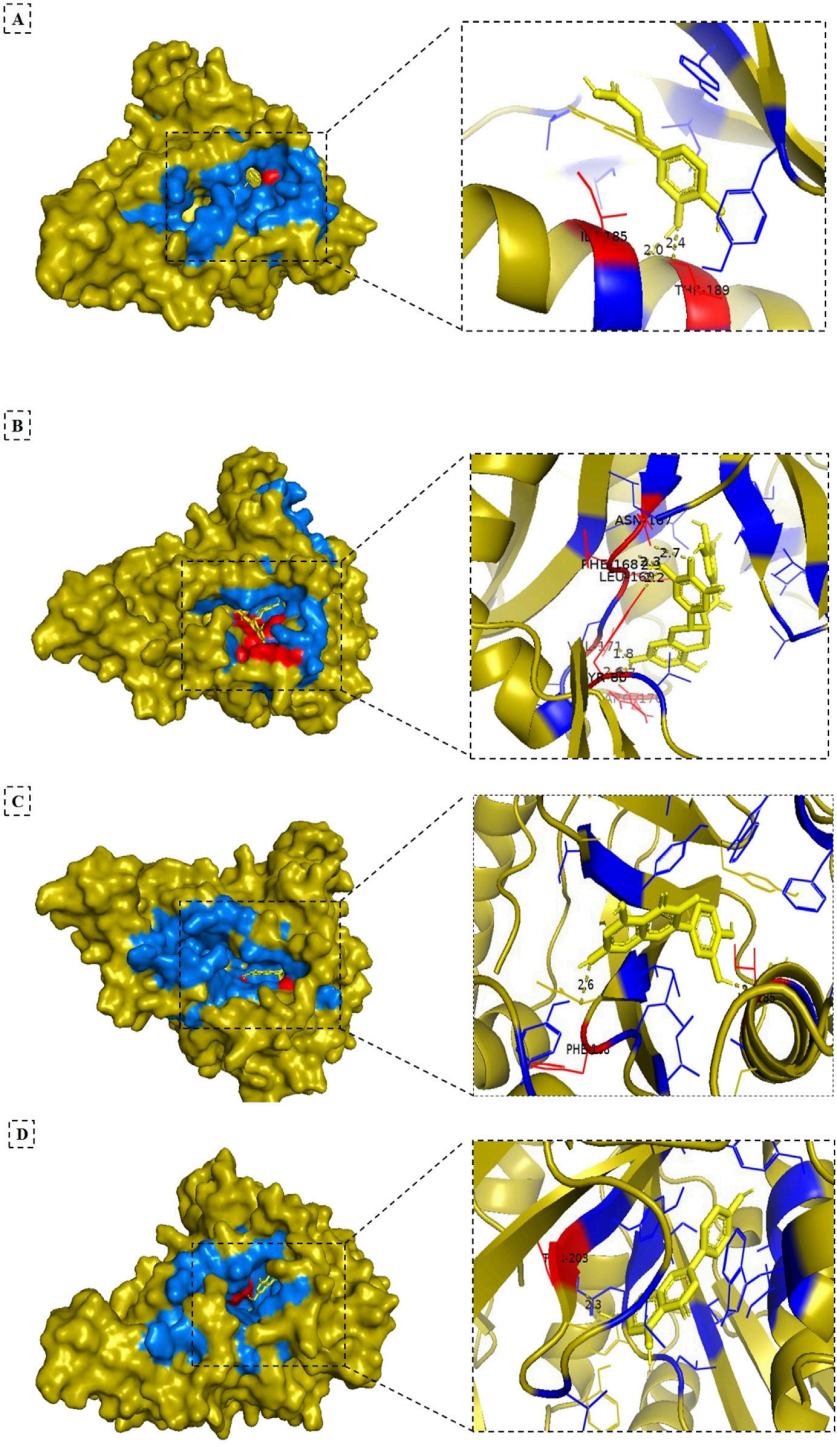
3D surface views (left) of the NMT ligand complexes, and cartoon structure views (right) of the binding pocket after docking. The olive orange color indicates the target protein (NMT), yellow denotes the ligand (compounds from *Artemisia absinthium* extract), red represents the hydrogen-bonding residues, and blue shows the hydrophobic interacted residues. Panels **(A–D)** show the complexes of NMT with caffeic acid, epigallocatechin gallate, quercetin, and apigenin, respectively.

Given the superior binding affinities and strong interactions of apigenin, quercetin, epigallocatechin gallate, and caffeic acid, these four compounds warrant further investigation in subsequent studies (Figure 3). Their significant binding characteristics and interactions with key residues suggest a higher potential for therapeutic applications against leishmaniasis. According to Carter et al. (2021), flavonols such as quercetin and green tea flavanols like epigallocatechin gallate have been shown to inhibit Leishmania arginase and modulate the host immune response, presenting promising antileishmanial effects with minimal toxicity to the host. These findings emphasize the potential of these compounds for future therapeutic strategies against leishmaniasis, making them the focus of our analysis moving forward.

**FIGURE 4:**
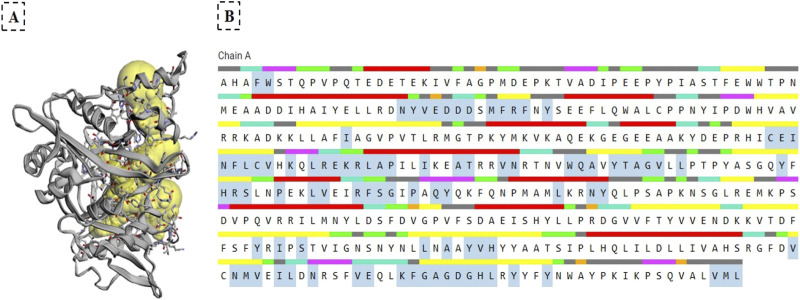
Identification of the NMT Binding Pocket by the CASTp Server. **(A)** Yellow spheres represent the binding sites of NMT. **(B)** Key amino acid residues within the active site are highlighted by cyan boxes. The colored lines above the amino acids indicate different structural regions: red represents α-helix, orange indicates β bridge, yellow shows strand, purple denotes 3_10_ helix, pink corresponds to π helix, green indicates turns, light green represents bends, grey shows coil regions, black indicates unstructured areas, underlined sections are annotated, and the amino acids within cyan boxes are displayed.

The binding pockets of NMT were identified with the CASTp server (Figure 4), Panel (A) displays yellow spheres marking the specific binding sites, while Panel (B) highlights the key amino acid residues within the active site using cyan-colored boxes. These residues play a crucial role in the molecular docking interactions (e.g., hydrogen bonding and hydrophobic contracts) between the phytochemicals and NMT. This structural analysis complemented the binding energy data (Table 4) and 3D visualizations (Figure 3) and provided critical insights into how these phytochemicals interact with NMT, bringing some clues on the molecular basis of their different therapeutic potentials.

### 3.6 Molecular dynamics simulations of the Apigenin-NMT complex

Due to its superior pharmacological and toxicological properties, as well as its favorable binding affinity, reflected by an estimated binding free energy of −9.6 kcal/mol, apigenin was selected for molecular dynamics (MD) simulation analysis to assess the stability and behavior of the apigenin-NMT complex. The binding free energy value of −9.6 kcal/mol indicates a strong interaction between apigenin and NMT, suggesting that apigenin has a high potential to effectively inhibit NMT activity. This observation is consistent with findings from Hou et al. (2022), who reported that various bioactive compounds, including polyphenolics similar to apigenin, exhibit strong enzyme-inhibiting properties. Specifically, Hou et al. highlighted the inhibitory effects of compounds from Perilla frutescens on enzymes like NMT, noting their significant pharmacological potential due to their ability to modulate posttranslational enzyme activity. These findings further support apigenin’s potential as a promising NMT inhibitor in therapeutic applications, especially in combating diseases like leishmaniasis where NMT plays a crucial role.

**FIGURE 5:**
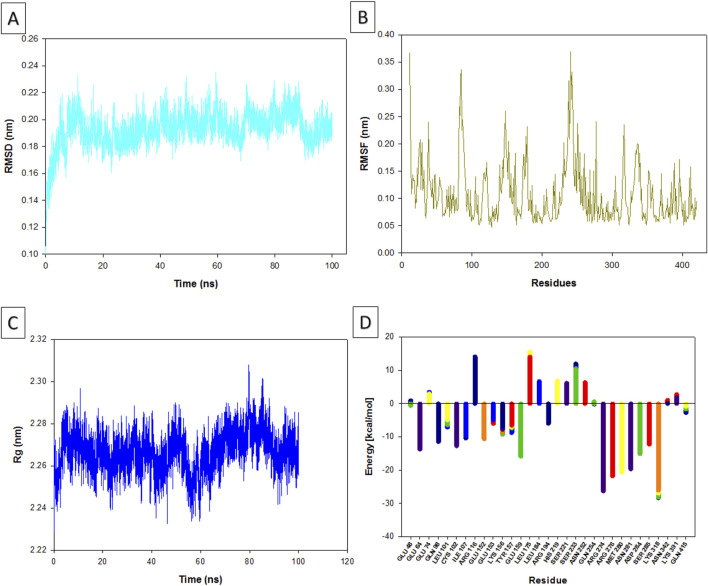
Results of the molecular dynamic analysis. Changes in the root mean square deviation (RMSD) values over time **(A)**. Root mean square fluctuation (RMSF) **(B)**. Changes in the radius of gyration (Rg) values over time **(C)**. Binding free energy calculations **(D)**.

The RMSD (Root Mean Square Deviation) values provide insight into how much the apigenin-NMT complex deviated from its original structure during the MD simulation (Figure 5A). Over the 100 ns simulation, the complex exhibited a low mean RMSD value of 0.04 nm, indicating exceptional structural stability with minimal deviation. This suggests that apigenin maintained a consistent binding pose within the active site of NMT throughout the entire simulation. This stability mirrors findings by Yang and Kar (2024), where MD simulations of small molecules against the Nipah virus demonstrated that compounds with low RMSD values tend to maintain stable interactions within their binding sites, enhancing their potential as inhibitors. In this context, stability in RMSD is often linked to a well-defined binding conformation, further implying apigenin’s potential as a robust NMT inhibitor. Frimurer et al. (2003) also emphasize the importance of accounting for ligand-induced conformational changes in target proteins and incorporating side-chain flexibility during docking simulations. They highlight that low RMSD values are indicative of stable ligand-protein interactions and serve as strong predictors of effective inhibitors in structure-based drug design. These findings collectively reinforce the idea that apigenin’s stable interaction with NMT could make it a potent therapeutic candidate for diseases like leishmaniasis.

The RMSF values provide insight into the flexibility of specific amino acid residues within the apigenin-NMT complex (Figure 5B). The observed low RMSF values (ranging from 0.05 to 0.35 nm) indicate that the interactions between NMT and apigenin are stable, with minimal fluctuations across the amino acid residues. This suggests that the ligand does not induce significant conformational changes in the protein, a crucial factor for maintaining the functional integrity of the protein. The stability reflected by these low RMSF values is essential for apigenin’s potential therapeutic efficacy as an inhibitor. Proteins often rely on maintaining a specific conformation for their functional activity, and significant flexibility could disrupt these critical interactions. On the other hand, higher RMSF values, which signify increased flexibility, often correlate with dynamic regions of the protein that are involved in various interactions, while lower RMSF values are indicative of more rigid and stable binding interactions. This rigidity is a key characteristic of effective inhibitors, as noted by Sharma et al. (2021), which further underscores apigenin’s potential as a robust NMT inhibitor.

The Rg (Radius of Gyration), which measures the overall compactness of the protein-ligand complex, remained stable in the range of 2.24–2.30 nm during the simulation (Figure 5C). This consistent range indicates that the apigenin-NMT complex retained its structural integrity throughout the MD simulation, suggesting that apigenin binds stably to NMT without inducing significant structural alterations. A stable Rg reflects consistent compactness of the complex, which is a key indicator of a favorable and stable interaction between the ligand and the target protein. The importance of Rg as an indicator of complex stability is supported by studies like Arooj et al. (2020), which analyzed protein–benzoic acid complexes and found that stable Rg values correlated with structural stability, particularly in complexes involving proteins like BSA and lysozyme. Similar to these findings, the Rg range observed in the apigenin-NMT complex suggests that apigenin binds to NMT in a manner that preserves the compactness and overall shape of the protein, further highlighting the stability and therapeutic potential of this interaction.

The molecular structure of apigenin (C₁₅H₁₄O₅) is characterized by multiple hydroxyl (-OH) groups, a catechol moiety, and a double bond, all of which significantly contribute to its ability to form hydrogen bonds with amino acid residues in NMT, particularly with threonine residues. These interactions enhance apigenin’s binding affinity and stability within the enzyme’s active site. Research has demonstrated that flavonoids possessing hydroxyl groups typically exhibit stronger binding interactions due to the formation of hydrogen bonds (Lantz et al., 2020). Moreover, the presence of the double bond in apigenin’s structure contributes to its rigidity and facilitates proper alignment within the active site of the enzyme, thereby strengthening its inhibitory potential (Xiao et al., 2023). This combination of structural features is critical for ensuring that apigenin maintains a stable and effective interaction with NMT. In addition, the aromatic rings in apigenin’s structure allow for π-π stacking interactions, which further stabilize the apigenin-NMT complex. Zhuang et al. (2019) emphasize that π-π stacking interactions are essential non-covalent forces that play a vital role in the design of drug delivery systems. These interactions enable effective drug loading into delivery vehicles without compromising the structural or functional integrity of either the drug or the delivery system, thereby maintaining their effectiveness and stability.

The calculated free energy values for individual residues in *L. major* NMT exhibit considerable variation, reflecting distinct energetic contributions that influence the stability of the apigenin-NMT complex (Figure 5D). Residues such as LYS 318 and ARG 274, with energy values ranging from approximately −26.098 to −28.364 kcal/mol and −23.899 to −26.331 kcal/mol, respectively, show strong stabilizing effects. These low energy values correlate with the observed low RMSF in the MD simulations, supporting the notion that apigenin’s binding to these residues significantly enhances complex stability. This stability is critical, as it suggests that interactions with residues like LYS 318 and ARG 274 help preserve the structural integrity of NMT, aligning with apigenin’s potential as an effective NMT inhibitor.

In contrast, residues with positive free energy values, such as ARG 116 (8.535–14.150 kcal/mol) and LEU 175 (13.988–15.593 kcal/mol), appear to contribute less to the stability of the apigenin-NMT interaction. The MD results, including low root mean square deviation (RMSD), RMSF, and stable radius of gyration (Rg) values, further highlight that apigenin selectively interacts with stabilizing residues rather than these destabilizing sites within the active site of NMT. Additionally, residues like GLU 64 and GLU 159, with negative energy values, contribute collectively to the overall stability of the protein structure. This stabilizing effect likely aids apigenin in maintaining the compactness of NMT, as evidenced by stable Rg values throughout the simulation. The consistent interactions between apigenin and these stabilizing residues provide a solid foundation for its inhibitory effect on NMT, as suggested by the free energy landscape and corroborated by MD analysis.

**FIGURE 6:**
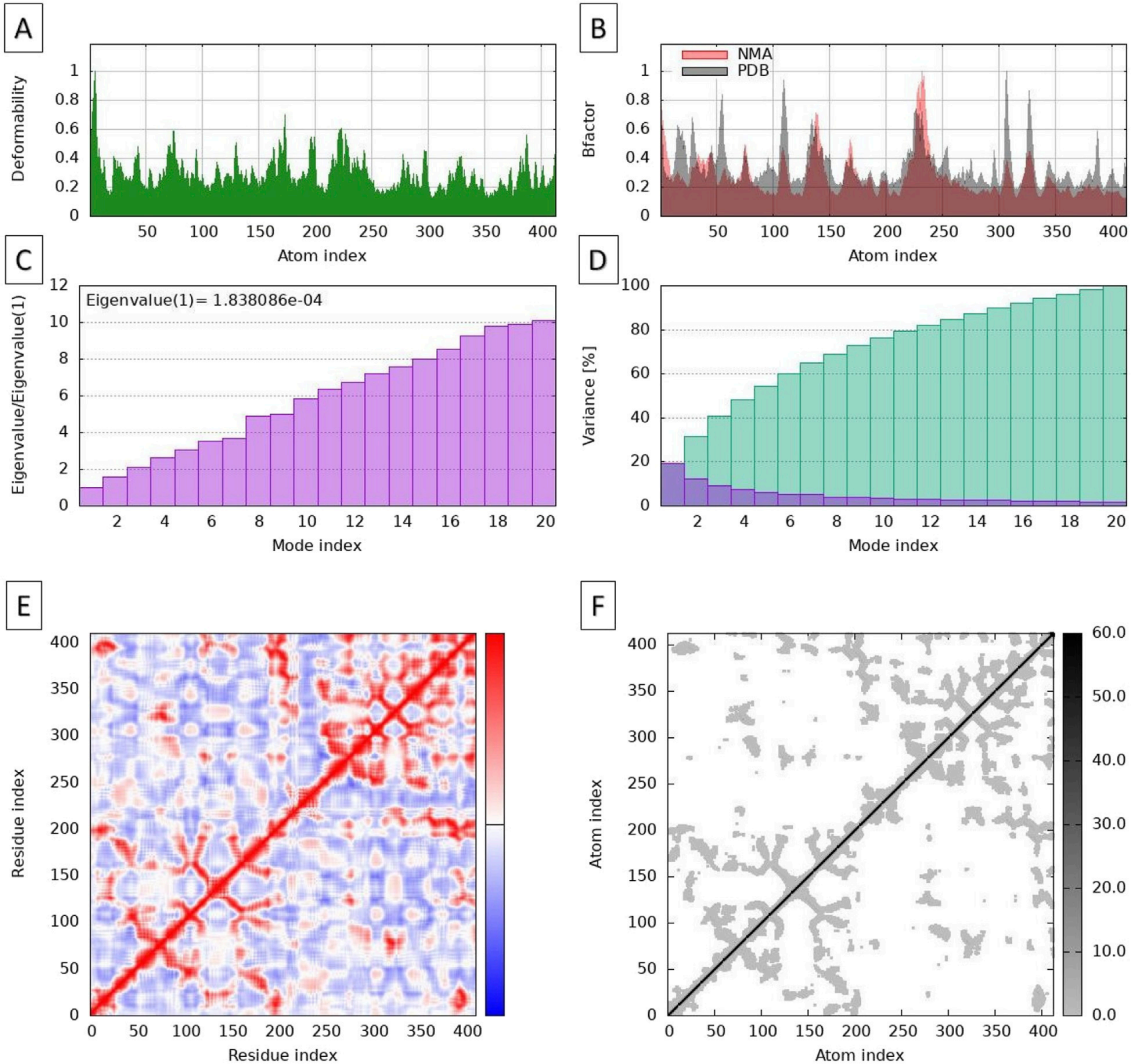
Molecular dynamic simulations, using normal mode analysis, of the NMT-apigenin complex. Deformability plots **(A)**, B-factor plots **(B)**, Eigenvalues **(C)**, Variance map **(D)**, Correlation matrix **(E)**, and Elastic network model **(F)**.

The Normal Mode Analysis (NMA) provided valuable insights into the flexibility and functional dynamics of the apigenin-NMT complex. The deformability plot (Figure 6A) illustrates regions within the protein that exhibit varying degrees of flexibility, which may be crucial for functional movements. The deformability data from the MD simulations show values ranging from very low (1.33E-03) to high (8.20E-03). Lower deformability values indicate rigidity, while higher values suggest regions of flexibility. Notably, higher deformability is observed at indices 1 to 6, reflecting more flexible regions, whereas a decline in deformability between indices 12 to 20 suggests greater stability in these regions. This stability likely corresponds to essential structural components that contribute to maintaining the integrity of the protein-ligand complex. The fluctuations observed around indices 60-70 highlight areas that may be significant for protein-ligand interactions. In contrast, the lower deformability values at indices 11, 19, 53, and 215 point to stable structural elements, such as alpha helices or beta sheets, which are known to be less flexible (Garzon et al., 2007).

The B-factor diagram provides insights into the thermal vibrations within the protein-ligand complex, with lower B-factor values indicating more rigid regions and higher values suggesting areas of greater flexibility (Figure 6B). The analysis of B-factors demonstrated a strong correlation between experimental B-factors (ranging from 10 to 50 Å^2^) and the calculated values derived from Normal Mode Analysis (NMA), with a Pearson correlation coefficient of 0.85. This strong correlation suggests that the dynamic behavior of the complex can be effectively modeled using NMA.

The experimental B-factors were obtained from the relevant field in the Protein Data Bank (PDB) file, while the calculated values were derived by multiplying the NMA mobility values by 8*π* ^2^. High mobility regions, such as residues 45-60, displayed calculated B-factors exceeding 40 Å^2^, which aligns well with the experimental observations. This correspondence indicates that these regions are indeed more dynamic, consistent with their role in facilitating interactions and conformational changes. However, some discrepancies were noted in specific areas, particularly in residues 15-25, where the experimental B-factor averaged 15 Å^2^ while the calculated value was significantly higher at 30 Å^2^. These differences may indicate variations in the actual dynamics of these residues compared to the model’s predictions, suggesting that further investigation into these specific regions is warranted to understand the factors influencing their conformational behavior.

The eigenvalue analysis of the apigenin-NMT complex provided insights into the vibration modes of the system, with higher eigenvalues indicating stiffer or less flexible regions (Figure 6C). In this analysis, the eigenvalue was calculated to be 1.838086 × 10⁻⁴, suggesting that the system exhibits significant flexibility in the analyzed modes. Each eigenvalue corresponds to the stiffness of the motion in that normal mode, directly relating to the energy required for structural deformation. Therefore, a lower eigenvalue indicates that deformation is easier, which aligns with the observed mobility in certain regions of the complex, suggesting these areas can undergo substantial conformational changes with minimal energy input.

The variance map (Figure 6D) provides a visual representation of the regions with greater atomic fluctuation. It illustrates the relationship between mode indices and variance, with cumulative variance (represented by green bars) increasing to reach 100% by mode index 14, effectively capturing the overall variance of the system. The individual variance (represented by purple bars) highlights the contributions of specific modes, indicating that modes 2 to 12 are particularly critical for understanding atomic fluctuations. Higher variance values reflect greater atomic movement, and the inverse relationship between variance and eigenvalues emphasizes that lower-frequency modes are more significant for atomic motion. Key modes, such as 2, 4, 6, 8, 10, 12, and 14, correspond to cumulative variances from 20% to 100%.

The covariance matrix (Figure 6E) further elucidates the coupling between pairs of residues within the protein. In this matrix, indices range from 0 to 400, with red areas indicating correlated motions, which suggest that residues in these regions move together. This correlation can be crucial for maintaining the structural integrity of the protein. For example, residues around indices 150 to 250 exhibit strong correlations, indicating functional interdependence. Conversely, the white areas represent uncorrelated motions, implying independent movement among those residues, while blue regions denote anti-correlated motions, where residues around indices 100 and 300 may exhibit opposite movements. The dense clustering of red in certain sections, particularly from indices 200 to 300, indicates areas of structural rigidity or functional interdependence, whereas the sparse blue regions suggest flexibility or adaptive responses in the protein’s dynamics. This comprehensive analysis underscores the intricate interplay between residues, which is vital for understanding the protein’s function and stability, as highlighted by James and Verkhivker (2014). Understanding these dynamics can inform future therapeutic strategies targeting the NMT enzyme.

The elastic network plot (Figure 6F) illustrates the connections between pairs of atoms within the apigenin-NMT complex, represented as springs. Each dot on the plot corresponds to a spring, and the color intensity of the springs indicates their stiffness: darker shades of gray represent stiffer connections, while lighter shades indicate more flexible ones. The diagonal line from the bottom left to the top right represents self-connections, where springs connect an atom to itself, which are inherently strong, with stiffness values reaching up to 60.0. Clusters of stiffer springs are notably observed in the regions around indices 150 to 250, suggesting that these atoms have stronger interactions, which are crucial for maintaining the structural integrity of the protein. In contrast, areas with lighter dots, particularly beyond index 300, indicate greater flexibility. This flexibility may facilitate conformational changes within the protein, allowing it to adapt functionally to various stimuli.

The elastic network analysis highlights the dynamic interplay of stiffness within the protein’s framework, emphasizing its functional adaptability as discussed by Dubanevics and McLeish (2022). Together, these analyses (Figures 5, 6) provide a comprehensive understanding of the structural stability, flexibility, and interactions within the apigenin-NMT complex. Such insights are essential for drug discovery and development, as they elucidate the complex molecular behavior that influences the effectiveness of potential inhibitors like apigenin. This detailed examination not only reinforces the potential therapeutic applications of apigenin but also guides future research in optimizing interactions for enhanced pharmacological outcomes.

## 4 Conclusion

This study examined the phytochemical profile, drug-likeness, toxicity risks, and molecular interactions of bioactive compounds derived from Artemisia absinthium. Among the key compounds identified—caffeic acid, ferulic acid, syringic acid, epigallocatechin gallate, vanillic acid, chlorogenic acid, kaempferol, quercetin, and apigenin—apigenin and caffeic acid demonstrated the most promising characteristics for leishmaniasis treatment. Apigenin, in particular, showed a strong inhibitory effect on Leishmania N-myristoyltransferase (NMT), with a binding energy of −9.6 kcal/mol and significant interactions with threonine residues 203 and 189. Drug-likeness analysis confirmed that most compounds met Lipinski’s rule of five, indicating favorable pharmacokinetic properties, while toxicity assessments revealed low-risk profiles for apigenin and caffeic acid. MD simulations reinforced the stability of the apigenin-NMT complex, with RMSD values of 0.04 nm, RMSF values ranging from 0.05 to 0.35 nm, and an Rg range of 2.24–2.30 nm. Normal mode analysis further supported the complex’s stability and flexibility, underscoring apigenin’s potential as a reliable NMT inhibitor. These findings highlight apigenin and caffeic acid as promising candidates for antileishmanial drug development, providing a foundation for future research aimed at optimizing these compounds for clinical applications.

## Data Availability

The raw data supporting the conclusions of this article will be made available by the authors, without undue reservation.
